# Constant force grinding controller for robots based on SAC optimal parameter finding algorithm

**DOI:** 10.1038/s41598-024-63384-2

**Published:** 2024-06-19

**Authors:** Chosei Rei, Qichao Wang, Linlin Chen, Xinhua Yan, Peng Zhang, Liwei Fu, Chong Wang, Xinghui Liu

**Affiliations:** 1 Nobot Intelligent Equipment (Shandong) Co., Ltd, Liaocheng, Shandong China; 2 School of Mechanical and Automotive Engineering, Liaocheng University, Liaocheng, Shandong China; 3 Department of Materials Physics, Saveetha School of Engineering, Saveetha Institute of Medical and Technical Sciences (SIMTS), Chennai, Tamilnadu India; 4 Science and Technology on Aerospace Chemical Power Laboratory, Hubei Institute of Aerospace Chemotechnology, Xiangyang, 441003 China

**Keywords:** Robots, Constant force grinding, Soft actor critic, Optimal parameter finding algorithm, Simulation model, Mechanical engineering, Engineering, Physics

## Abstract

Since conventional PID (Proportional–Integral–Derivative) controllers hardly control the robot to stabilize for constant force grinding under changing environmental conditions, it is necessary to add a compensation term to conventional PID controllers. An optimal parameter finding algorithm based on SAC (Soft-Actor-Critic) is proposed to solve the problem that the compensation term parameters are difficult to obtain, including training state action and normalization preprocessing, reward function design, and targeted deep neural network design. The algorithm is used to find the optimal controller compensation term parameters and applied to the PID controller to complete the compensation through the inverse kinematics of the robot to achieve constant force grinding control. To verify the algorithm's feasibility, a simulation model of a grinding robot with sensible force information is established, and the simulation results show that the controller trained with the algorithm can achieve constant force grinding of the robot. Finally, the robot constant force grinding experimental system platform is built for testing, which verifies the control effect of the optimal parameter finding algorithm on the robot constant force grinding and has specific environmental adaptability.

## Introduction

Traditional manual grinding not only has uneven grinding quality and low efficiency but also generates dust during the grinding process, which is hazardous to the physical and mental health of workers, while robotic grinding can replace manual grinding and achieve automation^1,2^. Maintaining a constant grinding force during robotic grinding not only ensures the accuracy and consistency of the surface quality of the workpiece but also reduces the wear and tear on the robot and end-effector^3^. Therefore, robotic constant force grinding has become a hot research topic.

Robot constant force grinding is divided into two stages: passive flexibility control and active flexibility control. Passive flexibility control passively changes the contact force by installing a passive suppleness device at the end of the robot. Wang et al. proposed a new type of passive suppleness control method for overcoming the influence of fluctuating normal grinding force on the precision grinding of nickel-based high-temperature alloy blade abrasive belts and proved through experiments that the method can improve the control accuracy of normal grinding force^4^. However, passive flexibility control does not have force feedback and cannot perform precise grinding force control, so active flexibility control using force sensors for force feedback has become the research focus. Active flexibility control mainly goes through three stages: impedance, adaptive, and intelligent control. Impedance control is adding a mass-damping-spring system at the robot's end, which establishes the relationship between the contact force and the end position. However, in practice, precise constant force control cannot be achieved solely through impedance control. Therefore Wang et al. proposed an adaptive variable impedance control algorithm with pre-PD adjustment to realize the soft grinding and polishing of complex surfaces, and the stability and convergence of the force control algorithm were proved by comparative experiments^5^. Li, based on traditional impedance control, proposed a position-based force-tracking adaptive impedance control strategy to improve the complex surface grinding quality of aero engine parts grinding quality of aero-engine^6^.

On the other hand, adaptive control can adapt to changes in the environment. Zhao et al. proposed an adaptive constant force controller based on stiffness estimation, which realizes constant force grinding of unknown workpiece contours and ensures grinding consistency^7^. Zhang et al. proposed a trajectory compensation method based on the collaborative kriging spatial interpolation method to reduce the grinding trajectory deviation caused by the robot's absolute positioning accuracy and the trajectory compensation method based on the one-dimensional force tracking method. Meanwhile, an adaptive iterative constant force control method based on a one-dimensional force sensor is proposed to improve the processing quality and efficiency of robot belt grinding^3^.

However, the adaptive capacity of adaptive control is related to the intrinsic parameters, and if the parameters exceed the range of intrinsic parameters, the system will be out of balance. With the continuous development of artificial intelligence, more and more intelligent algorithms are applied to robot constant force control^8^. Such as fuzzy control, neural networks, and reinforcement learning, each of which has its advantages. The fuzzy control algorithm has better robustness; when there are disturbances or parameter changes in the system, the fuzzy control algorithm still has a better control effect. Zhang et al. developed a hybrid force/position anti-disturbance control strategy based on fuzzy PID control to improve the grinding quality of aerospace blades^9^. Shen et al. proposed a fuzzy-based adaptive impedance control that can grind or polish workpieces of different materials with constant contact force^10^. The neural network algorithm is robust, fault-tolerant, and can adequately approximate any complex nonlinear relationship. Conventional PID makes it challenging to realize the problem of complex parameters and nonlinear characteristics; Zhu et al. developed a PID controller based on a fuzzy neural network algorithm for simultaneous trajectory and contact constant force tracking, which proved the effectiveness through simulation results^11^. Hamedani et al. proposed an intelligent impedance control strategy based on wavelet neural networks to adapt the impedance parameters according to the variable environment, and the experimental results proved that the method has better force tracking performance than the general impedance with constant parameters^12^. Reinforcement learning has apparent advantages in the direction of complex system control, solving nonlinear problems, and finding optimal parameters, so it is widely used in the fields of neurobiology^13^, autonomous driving^14^, and robotics^15^. Zhang et al. proposed an online optimization method for constant force grinding controller parameters based on deep reinforcement learning Rainbow. The technique can optimize the control parameters online while converging stably to solve the constant force control problem in the grinding process^16^. Zhang et al. proposed a reinforcement learning-based machine manpower control algorithm to solve the problem that the contact force of the robot end-effector is challenging to keep constant when the robot is tracking an unknown curved workpiece^17^.

Since the conventional PID controller cannot control the robot to stabilize for constant force grinding under environmental changes, a compensation term needs to be added to the conventional PID controller. To solve the problem that the compensation term parameters are difficult to obtain, an optimal parameter finding algorithm based on SAC (Soft-Actor-Critic) is proposed, including training state action and normalization preprocessing, reward function design, and targeted deep neural network design. The algorithm is used to find the optimal compensation term parameters and applied to the PID controller to accomplish the compensation through the robot's inverse kinematics for constant force grinding control. The innovations of this paper are as follows.

① An optimal parameter finding algorithm based on SAC is proposed to obtain the optimal controller compensation term parameters, and a deep neural network is utilized to fit a model of the relationship between the training grinding force and the end-effector compensation displacement, and the compensation is accomplished by using the robot inverse kinematics. The deep neural network that completed the training was used as a robot constant force grinding controller.

② A robot grinding simulation platform with perceptible force information was built to verify the feasibility and adaptability of the robot constant force controller.

③ The robot constant force grinding experimental system platform is built, and bench tests are conducted to verify the control effect of the SAC-based optimal parameter finding algorithm on robot constant force grinding.

The main contents of this paper are organized as follows. In the first part of this paper, the mechanical model of the end-effector will be designed. In the second part, a specific analysis of the optimal control parameter solution algorithm based on SAC for the compensation term will be carried out. The experimental design of the robotic grinding simulation platform with force information sensing will be designed in the third part. The bench test of the robotic grinding system platform will be carried out in the fourth part, and the conclusion will be given in the fifth part.

## Mechanical modeling of robotic end-effector

**Figure 1:**
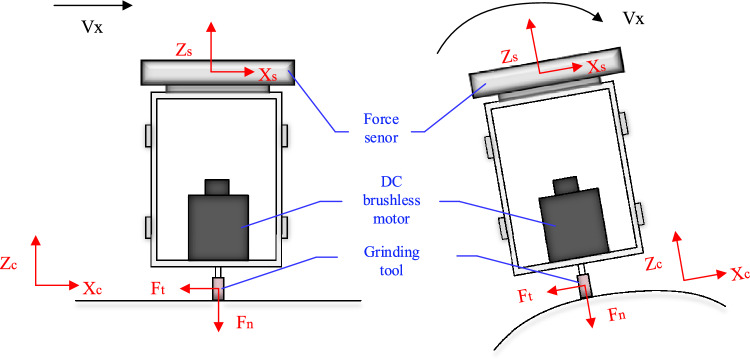
End-effector contact force analysis.

The force analysis of the end-effector when the robot is grinding flat and curved surfaces, respectively, as shown in Fig. 1. The positional relationship between the sensor coordinate system {S} and the workpiece coordinate system {C} is established according to the model during grinding. The origin of the sensor coordinate system is the geometric center of the sensor, the Z-axis direction is the axial direction of the sensor, the X-axis direction is the radial direction of the sensor, and the force in the Y-axis direction is determined according to the right-hand rule of the coordinate system. The origin of the workpiece coordinate system is the geometric center of the workpiece, the X-axis direction is the radial direction of the workpiece, the Z-axis direction is the axial direction of the workpiece, and the Y-axis direction is determined according to the right-hand rule of the coordinate system. The X-axis of the two coordinate systems is kept parallel during grinding.

Assuming that *F* and *G* are the tangential and normal forces of grinding in the coordinate system of workpiece, respectively, and M and N denote the forces that transfer *F* and *G* to the sensor's coordinate system, then $F_{t^{\prime}} = F_{t}$, $F_{{{\text{n}}^{\prime}}} = F_{n}$. When a grinding operation is performed, the normal force $F_{n}$ is the main object that affects the grinding effect.

Since the force sensor is mounted between the end of the robot and the grinding tool, its measured value $F_{s}$ includes not only the normal grinding force $F_{n}$ at the grinding end but also its own gravity $G$ and inertia force $F_{l}$ , as shown in Eq. (1), and therefore needs to be gravity compensated.

1 $$ F_{s} = F_{n} + G + F_{l} $$

Since the grinding process is at a constant speed and the acceleration is zero, the inertial force $F_{l}$ is very small and can be ignored. Here, the primary consideration is the measured value of the sensor for gravity compensation. The grinding robot end-effector is manually adjusted to a vertical downward position without contact with the grinding workpiece, at which point $F_{n}$ is 0. As the direction of gravity $G$ is always vertical downwards, opposite to the z-axis direction of the base coordinates, it can be expressed as ${}^{b}F_{G} = [0,0, - G_{T} ]^{T}$ under the base coordinates. When the robot arm changes position, the value of the base coordinates can be converted to the value of the sensor coordinates using the rotation matrix ${}_{b}^{s} R$, i.e.${}^{s}F_{G} = {}_{b}^{s} R \times {}^{b}F_{G}$, where ${}^{s}F_{G}$ is the influence value of gravity under the sensor coordinate system. So the grinding value at the sensor seated ${}^{s}F_{n}$ is the measured value minus ${}^{s}F_{G}$ , i.e.${}^{s}F_{n} = F_{s} - {}^{s}F_{G}$ .

## Research on optimal parameter finding algorithm based on SAC

To solve the problem that the compensation term parameters are difficult to obtain in the traditional PID controller, a deep reinforcement learning algorithm is introduced to find the optimal compensation term parameters. Deep reinforcement learning does not need to adjust the controller parameters artificially, but obtains the optimal compensation term parameters through self-learning the algorithm itself.

### Design of the PID controller

The target force $F_{d}$ is compared with the current normal force $F_{n}$, and the difference between the two is converted into a compensating displacement of the end-effector by the force controller, which is adjusted by the robot inverse kinematics, thus indirectly controlling the contact force between the robot and the environment. The PID controller is chosen to model the relationship between the robot end force error and the robot control parameters due to its simple structure and ease of tuning the parameters.

As the grinding process progresses, the external environment may change, and to better accommodate such changes, a compensation amount is added to the conventional force controller with the equation shown in (2).

2 $$ \begin{aligned} e_{f} \left( t \right) = & F_{n} \left( k \right) - F_{n} \Delta \left( t \right) \\ = & k_{p} ef\left( t \right) + k_{d} ecf\left( t \right) + \delta_{k} \left( t \right){\text{sgn}} (ecf\left( t \right)) + \xi_{k} \left( t \right){\text{sgn}} (ef\left( t \right))\Delta y\left( t \right) \\ = & \Delta y\left( {t - 1} \right) + \Delta \left( t \right) \\ \end{aligned} $$

where $ef(t)$ is the error of normal force and target normal force in time period t; $ecf(t)$ is the rate of change of force error in time period t; $k_{d}$, $k_{p}$ are the fixed scale parameters, the fixed scale parameter can make the robot contact with the workpiece in the process of avoiding robot overload;$\delta_{k}$,$\zeta_{k}$ are the compensation amount parameters; $sgn$ is the step function, its size is − 1 or 1. When the fixed scale parameter is set, the compensation term parameter is obtained by reinforcement learning.

**Figure 2:**
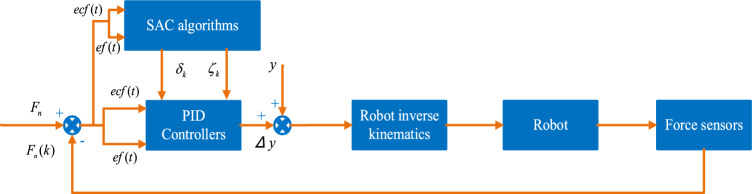
Schematic diagram of robot constant force grinding control based on SAC's optimal parameter finding algorithm.

Manually adjusting the compensation amount requires a lot of time and effort; however, reinforcement learning intelligently acquires the final control parameters through its self-learning capability without the need to manually adjust the compensation parameters, so reinforcement learning is used to learn the control parameters during the grinding process and complete the constant force grinding work. Reinforcement learning aims to maximize the expectation of the cumulative reward. In robot constant force grinding control, the SAC algorithm continuously iterates on the strategy to improve the selection of control parameters, and the optimal control parameters are defined when the expectation of the cumulative maximum reward is maximized. The grinding control schematic is shown in Fig. 2. Firstly, the normal force $F_{n}$ is read by the force sensor, the current read normal force and the target force $F_{d}$ are compared, the force error, the rate of change of the force error, and the controller compensation parameter, which is searched by the SAC algorithm are inputted into the force controller, and the robot's inverse kinematics accomplishes the output of the compensated displacement and the compensation.

### Introduction to the SAC algorithm

**Figure 3:**
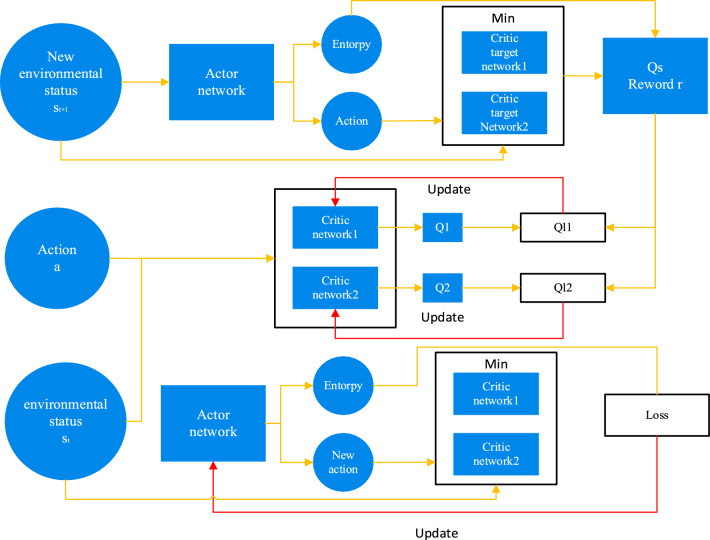
Structure of the SAC algorithm.

The SAC algorithm is formed based on the Actor-Critic network architecture, in which the SAC algorithm consists of an Actor network, two Critic networks, and two Critic target networks. The Critic network evaluates the action chosen by the actor network in the current state, while the Actor-network improves the desired action based on the Critic's evaluation of the activity, saving the $\{ s_{t} ,a_{t} ,s_{t + 1} ,r\}$ in the experience pool, which is used to update the parameters of the network. The SAC algorithm structure is shown in Fig. 3.

As shown in the figure above, the Critic network is updated first, when the environment state $s_{t}$ and the action $a_{t}$ selected under the environment state $s_{t}$ enter the Critic network, the two networks output different *Q* values, respectively, *Q* values represent the value evaluation of the action $a_{t}$ selected under the environment state $s_{t}$ , using the action entropy generated by the Actor network, the action entropy generated by the Critic target network $Q_{s}$ and the reward $r$ to reverse the update the Critic network, $Q_{s}$ serves to allow the Critic network to consider both the action entropy and the reward $r$; the Actor network is then updated using the KL scatter to evaluate the action selected by the Actor network and the Q value generated by the Critic network.

#### Calculation of action entropy

Usually, the goal of reinforcement learning is to maximize cumulative reward, as shown in Eq. (3).

3 $$ \pi * = \arg \max {\rm E}\left[ {\sum\limits_{t} {r(s_{t} ,a_{t} ))} } \right] $$

In contrast, the objective of the SAC algorithm is to maximize the cumulative reward with entropy, as shown in Eq. (4).

4 $$ \pi * = \arg \max {\rm E}\left[ {\sum\limits_{t} {r(s_{t} ,a_{t} ) + \alpha H(\pi ( \cdot |s_{t} ))} } \right] $$

where $\alpha$ is the temperature coefficient that determines the importance of entropy relative to the reward, and thus the degree of randomness of the control strategy; *H* is the entropy function, where $s_{t}$ ,$a_{t}$ are the state at moment *t* and the action selected in the state at time t respectively*; r* is the reward obtained.

The entropy function is calculated as shown in Eq. (5).

5 $$ H(\pi ( \cdot |s_{t + 1} )) = - {\rm E}_{a} \log \pi (a_{t + 1} |s_{t + 1} ) $$

where $s_{t + 1}$ is the state at the next moment of *t*; $a_{t + 1}$ is the action selected by the state at the next moment of *t*; $\pi (a_{t + 1} |s_{t + 1} )$ is the probability of taking the action $a_{t + 1}$ in the state $s_{t + 1}$.

The action value function for the Critic target network $Q_{s}$ shown in Fig. 3 is calculated from the action entropy and the action taken by the system at the next moment, and is calculated as shown in Eq. (6).

6 $$ \begin{gathered} Q_{s} = r + \lambda (V(s_{t + 1} )) \\ = r + \lambda (Q_{\pi } (s_{t + 1} ,a_{t + 1} ) + \alpha H(\pi ( \cdot |s_{t + 1} ))) \\ = r + \lambda (\mathop {\min }\limits_{j = 1,2} Q_{{\phi_{tj} }} (s_{t + 1} ,a_{t + 1} ) - \alpha \log \pi (a_{t + 1} |s_{t + 1} )) \\ \end{gathered} $$

where $V(s_{t + 1} )$ is the state value function under the state $s_{t + 1}$ , $\phi_{tj}$ is the parameter for the two Critic target networks, $\min Q_{{\phi_{tj} }} (s_{t + 1} ,a_{t + 1} )$ is the minimum value at the output of the two Critic target networks, which prevents overestimation of the estimated value.

#### Calculation of loss values for Critic's network

The SAC algorithm contains two identical Critic reviewer networks with the same structure as the Critic reviewer target network. In the algorithm, the two Critic reviewer networks are evaluated separately for the selected action and the difference with $Q_{s}$ is calculated, and this difference is the loss value, which is calculated as shown in Eq. (7).

7 $$ L(\phi_{i} ,D) = E_{{(s_{t} ,r,s_{t + 1} ,a_{t} )\sim D}} \left[ {(Q_{{\phi_{i} }} (s_{t} ,a_{t} ) - Q_{s} (s_{t + 1} ))^{2} } \right],i \in \{ 1,2\} $$

where $(s_{t} ,r,s_{t + 1} ,a_{t} )\sim D$ is the variable extracted from the experience pool D; $\phi_{i}$ is the weight of the *ith* Critic network; $Q_{{\phi_{i} }} (s_{t} ,a_{t} )$ is the evaluation of the *ith* network taking the action $a_{t}$ under the environmental state $s_{t}$ using the weight $\phi_{i}$.

#### Updates to the actor network

After the Critic reviewer network is continuously updated, its level of evaluating the Actor network for the selected action increases and the evaluation of the value of $Q_{s}$ will be close to the true value, using the KL scatter to update the Actor network, see Eq. (8).

8 $$ \mathop {\max }\limits_{\theta } E_{s\sim D} \left[ {\mathop {\min }\limits_{j = 1,2} Q_{{\phi_{j} }} (s_{t} ,a^{\prime}_{t} ) - \alpha \log \pi_{\theta } (a^{\prime}_{t} |s_{t} )} \right] $$

where $\theta$ is the weight of the Actor network and $\mathop {\min }\limits_{{\phantom{a}}} Q_{{\phi_{j} }} (s_{t} ,a^{\prime}_{t} )$ is the minimum value selected at the output of the two Critic reviewer networks, which prevents overestimation.

#### Updates to Critic's target network

The Critic target network is updated by scaling the parameters $\rho$ as shown in Eq. (9).

9 $$ \phi_{ti} \leftarrow \rho \phi_{ti} + (1 - \rho )\phi_{i} ,i \in \{ 1,2\} $$

The SAC algorithm pseudo-code is shown in Table 1.

**Table 1:** SAC algorithm pseudo-code.

SAC algorithm
1: Initialization parameters
2: Initialize Critic Network parameters, Critic Target Network parameters and Actor Network parameters
3: Emptying the experience pool D
4: Start the cycle M times
5: Start of cycle N steps
6: Selection of actions according to strategy $a_{t} \sim \pi_{\varphi } (a_{t} |s_{t} )$
7: Execute the action $a_{t}$ to obtain the next status $s_{t + 1}$ and the reward $r_{t}$
8: Store $a_{t}$ ,$s_{t}$ ,$s_{t + 1}$ ,$r_{t}$ in experience pool D
9: End of the cycle
10: Start the cycle L steps
11: Selecting quantitative data from the experience pool D to save to B $B = \{ (s_{t} ,r,s_{t + 1} ,a_{t} )\}$
12: Calculate the estimate of the Critic target network for Q $Q_{s} = r + \lambda (\mathop {\min }\limits_{j = 1,2} Q_{{\phi_{tj} }} (s_{t + 1} ,a_{t + 1} ) - \alpha \log \pi (a_{t + 1} |s_{t + 1} ))$
13: Update Critic network parameters $\nabla_{{\phi_{i} }} \frac{1}{|B|}\sum\limits_{(st,at,st + 1,r) \in B} {(Q_{{\phi_{i} }} (s_{t} ,a_{t} ) - Q_{s} (s_{t + 1} ))^{2} ,i \in \{ 1,2\} }$
14: Update Actor network parameters $\nabla_{\theta } \frac{1}{|B|}\sum\limits_{s \in B} {\left[ {\mathop {\min }\limits_{j = 1,2} Q_{{\phi_{j} }} (s_{t} ,a^{\prime}_{t} ) - \alpha \log \pi_{\theta } (a^{\prime}_{t} |s_{t} )} \right]}$
15: Update Critic target network parameters $\phi_{ti} \leftarrow \rho \phi_{ti} + (1 - \rho )\phi_{i} ,i \in \{ 1,2\}$
16: End of full cycle

### Design of SAC-based optimal parameter finding algorithm

In the SAC algorithm environment, the Agent is the robot, and the environment is the robot grinding environment where force information can be sensed.

In this SAC-based constant force grinding control algorithm, the state *s is* designed as the normal force error $ef$ and the rate of change of the normal force error $ecf$.

10 $$ s = [ef,ecf] $$

The output actions are the force controller compensation parameters $\delta_{k}$ and $\zeta_{k}$.

11 $$ a = [\delta_{k} ,\zeta_{k} ] $$

#### Design of the reward function

The goal of training is to enable the current normal force to reach the normal force, and the smaller the difference between the current normal force and the target normal force, the higher the reward is obtained. Therefore, the reward function used in designing the deep reinforcement learning SAC algorithm for constant force grinding control of a robot can be described as:

12 $$ r = - \sqrt {ef(t)^{2} } = - \sqrt {(F_{n} - F_{n} (k))}^{{_{2} }} $$

#### Normalized preprocessing of training data

In order to accelerate the convergence of SAC model, the data input to the model is normalized. The input state force error and force error rate and controller compensation parameters of the deep neural network are divided by the corresponding upper limits, respectively, so that each of these elements has a value domain of [− 1, 1] before entering the algorithm training; the normalised input state quantity is noted as *s_ norm* and the normalized output action is indicated as *a_ norm*.

#### Design of SAC neural network

According to the needs of the algorithm, the Actor and Critic networks in the SAC neural network are designed separately, and the Critic network consists of two target networks and two prediction networks.

**Figure 4:**
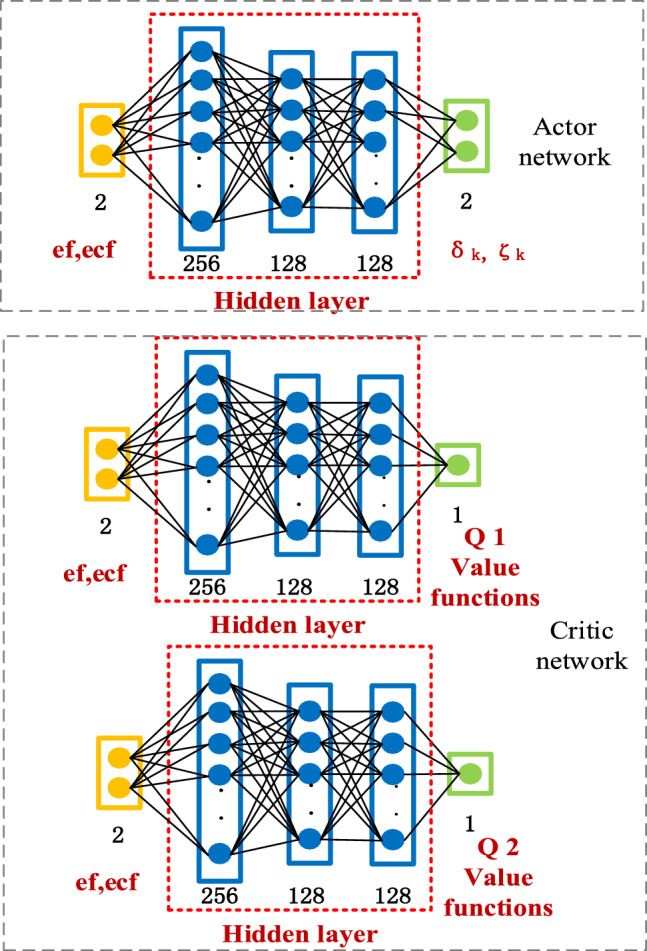
Structure of SAC neural network.

The structure of the Actor network has four layers; the number of nodes in the hidden layer is 256, 128, and 128, respectively, and the output is the probability distribution of the action and the action entropy, the activation function used between the hidden layers is Relu. The number of nodes in the hidden layers is 256, 128, 128, and the output is the Q-value, and the activation function used between the hidden layers is Relu. The structure of the SAC neural network is shown in Fig. 4.

The Critic network evaluates the value of the action under the current normal force error and corrects the parameters of the compensation term selected by the Actor network according to the critics' evaluation. The SAC algorithm requires only the Actor network output at the time of inference, and subsequent updates to the network require the data stored in the memory pool D $(s_{t} ,a_{t} ,r,s_{t + 1} )$.

## Simulation experiment

Coppeliasim is selected as the simulation software platform. Coppeliasim contains many encapsulated robots for easy use and supports Bullet and other physical engines. The physical machines enable the established simulation model to have mechanical properties and simulate the natural environment more accurately. At the same time, CoppeliaSim includes a remote API that allows communication with languages such as Python, solving the problem that the Lua language built into CoppeliaSim cannot build neural networks and enabling co-simulation. In addition, Coppeliasim provides an inverse kinematics solution module based on the numerical iterative solution, which can realize the function of directly outputting and executing the control action of the end tool pose by the grinding force control algorithm.

**Figure 5:**
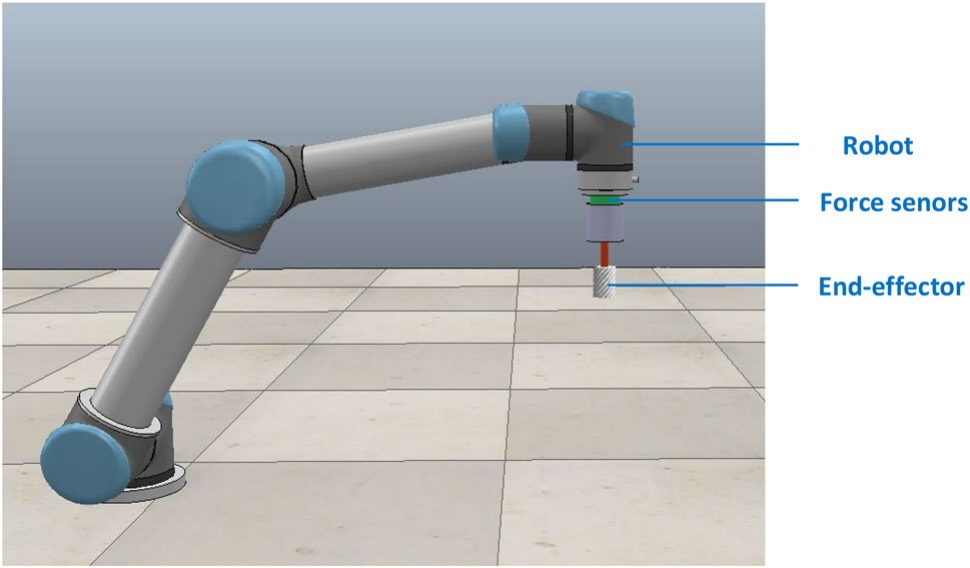
Force-aware robot grinding simulation model.

The sensible force information robot uses a Franka robot encapsulated in the simulation platform. The six-dimensional force sensor is mounted at the end flange position of the robot and is used to obtain the grinding force and grinding torque in the x, y, and z axes of the sensor. Then, set the mechanical characteristics, set the rotary axis to drive the end grinding tool for grinding operations, and install the end grinding actuator with the completed settings under the robot end six-dimensional force sensor to become part of the robot grinding simulation platform. It should be noted that the robot end flange, the six-dimensional force sensor, and the end grinding actuator need to be under the same Z coordinate. The perceptible force information robot grinding simulation model is shown in Fig. 5.

**Figure 6:**
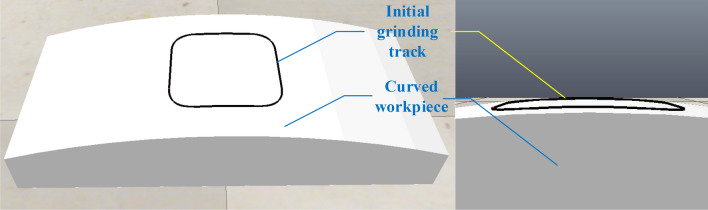
Surface workpiece and grinding initial trajectory.

The surface workpiece is imported into Coppeliasim, and its properties are set, and the tutorial sets the initial trajectory of grinding to make the robot end move along the initial trajectory. The plan view of the workpiece and the initial trajectory are shown in Fig. 6.

**Figure 7:**
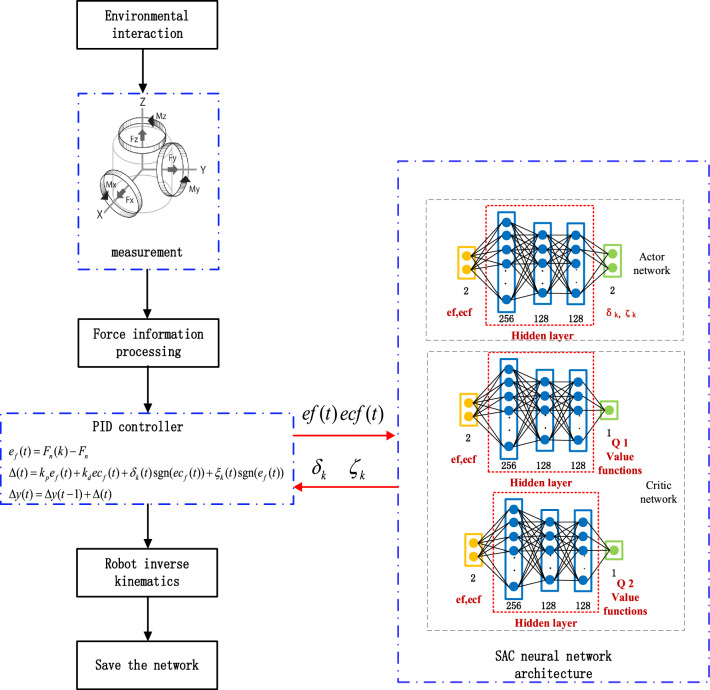
Flowchart of constant force grinding control based on SAC optimal parameter finding algorithm.

This simulation only investigates the processing of grinding. Before the experiment starts, the end-effector of the mobile robot is controlled to be above the initial point of grinding so that the end-effector is in contact with the grinding workpiece and the six-dimensional sensor acquires the initial force information so that the training experiment can be carried out. The flow chart of the constant force grinding control based on the SAC algorithm is shown in Fig. 7.

The target normal force is first set, followed by initialization of the robot position, starting the robot to interact with the environment to generate the grinding force and record the data; after force information processing, the force error $ef$ and force error rate $ecf$ are input into the neural network structure of the SAC algorithm, the output is the force controller compensation parameters after the force controller to get the compensation displacement of the robot end-effector, through the robot inverse kinematics to complete the compensation. The cycle continues until the end of the training, and the neural network is saved after the training.

The SAC algorithm neural network structure trains the robot for constant force grinding control. The total number of training sessions is set to 100, and each training session consists of 200 training steps, during which the robot and the workpiece interact, and the data obtained from the interaction is stored in the experience pool in a time series. The trained model needs to satisfy the deep network structure to converge to a steady state.

The trained neural network structure is used for finding the optimal parameters of the machine manpower controller; the inputs are the normalized force error $ef$ and the force error rate $ecf$, and the outputs are the compensation parameters $\delta_{k}$ and $\zeta_{k}$ of the force controller. The compensation is achieved by controlling the robot's joint angles obtained through the inverse kinematics of the robot. The specific parameters of the SAC algorithm are shown in Table 2.

**Table 2:** SAC algorithm parameter table.

Parameters	Value
Discount factor (γ)	0.9
Actor network learning rate	0.001
Critic network learning rate	0.0001
Number of batches	64
Tau	0.125
Alpha	0.2
*k*_*p*_	0.00002
*k*_*d*_	0.01
Mass of end-effector M	0.3 kg

**Figure 8:**
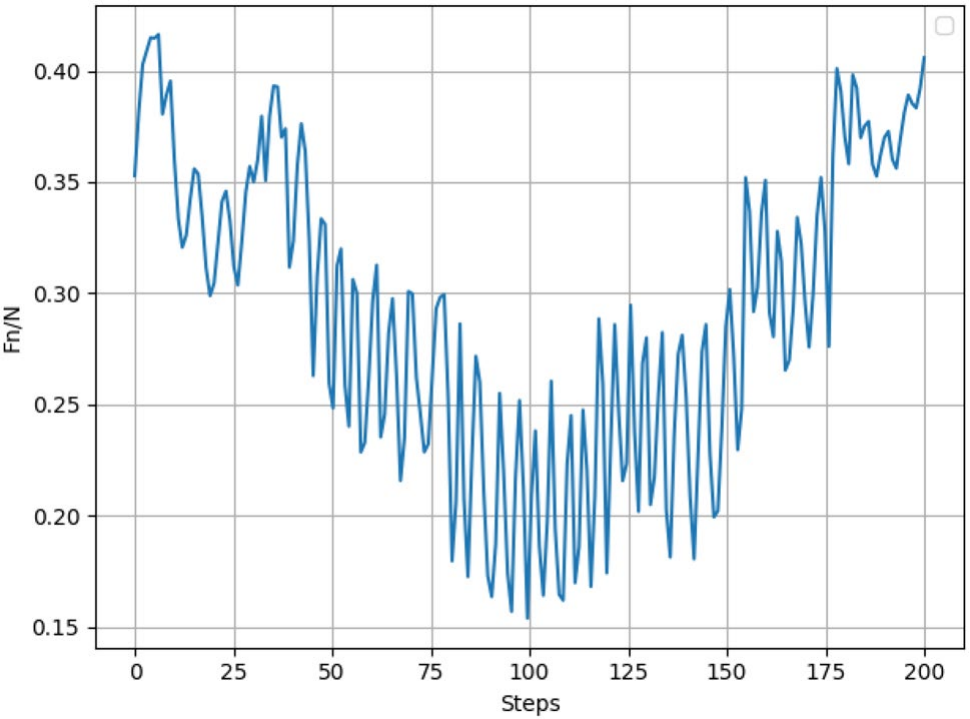
Variation of normal force using fixed parameters.

The SAC algorithm-based constant force grinding control for robots is used for robotic grinding of surfaces. Firstly, for comparison purposes, the force controller compensation parameters were not obtained using the SAC algorithm but rather using fixed compensation parameters. Figure 8 shows the variation of the normal force using the fixed compensation parameters $\delta_{k}$ = 0 and $\zeta_{k}$ = 0.2.

In the SAC algorithm-based robot constant force grinding, the system state is normal force error and normal force error rate, so only the normal force change needs to be observed. The graph shows that when using fixed compensation parameters, the value of normal force is unstable, fluctuates more, and cannot be stabilized to converge to the target normal force, which cannot meet the grinding requirements and force control is needed.

**Figure 9:**
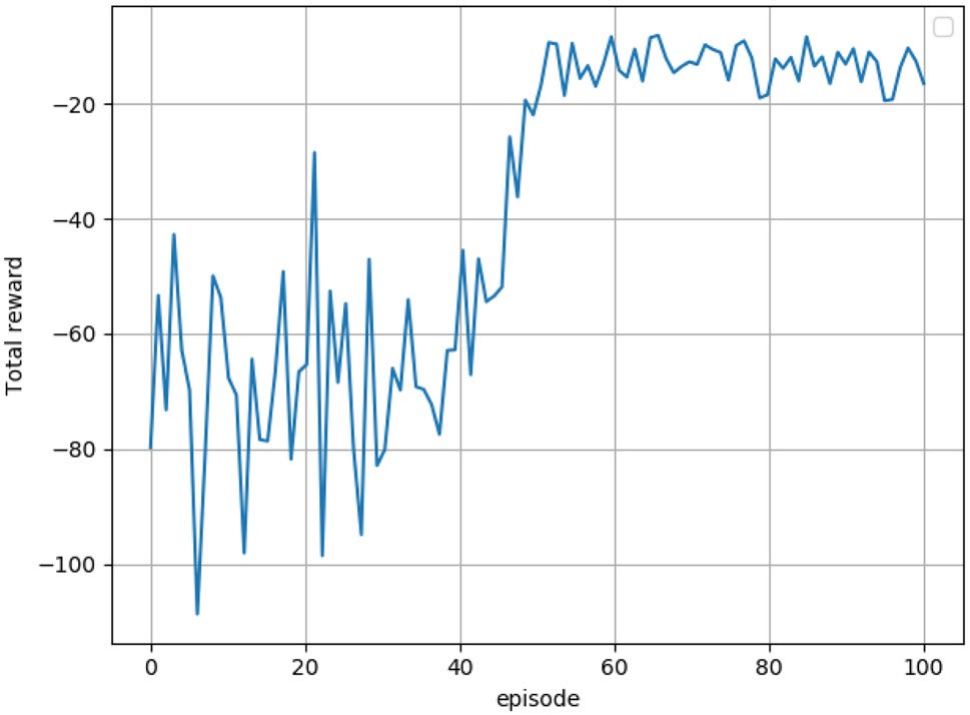
Total reward diagram for the SAC algorithm for sanding surface control.

The training method for the constant force sanding control was executed according to Fig. 7. After the deep neural network converged and saved, the total reward graph of the robot grinding surface control obtained at the end of the training is shown in Fig. 9. From the graph, it can be seen that the total reward kept fluctuating before the training times of 40, when the SAC algorithm was continuously trial and error, and after the training times of 40, the total reward value kept increasing as the training times improved and finally stabilized at around − 10. The results show that the gap between the current normal force and the target normal force is shrinking and gradually approaching the target polished force, and eventually fluctuates above and below the target normal force, indicating that the trained deep neural network model has converged to a stable state.

**Figure 10:**
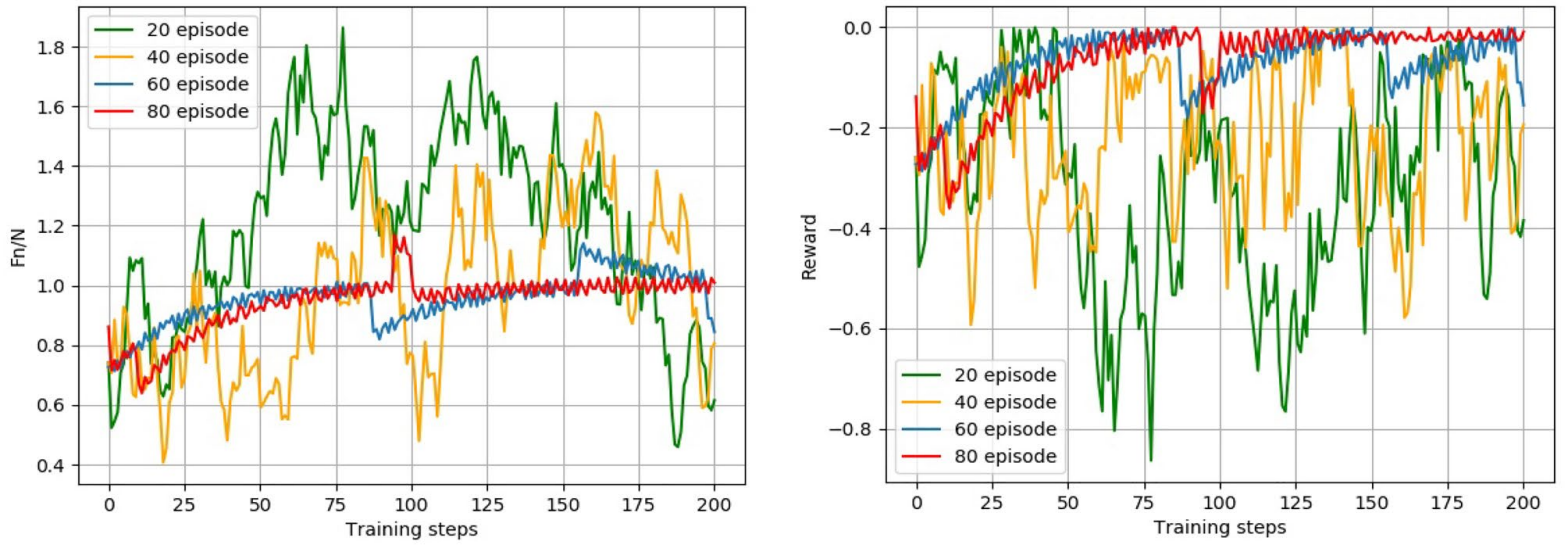
Iteration diagram of the SAC algorithm normal force and reward function.

The reward and normal force diagrams with 20, 40, 60, and 80 training sessions were selected for comparison, and the comparison is shown in Fig. 10.

The green, yellow, blue, and red curves in the graph represent the change in normal force and reward value for the 20th, 40th, 60th, and 80th training sessions, respectively. In the 60th and 80th training sessions, the normal force quickly reached the target normal force and maintained fluctuations above and below the target normal force, and even if there were sudden changes in the normal force, it could quickly recover to above and below the target normal force, indicating that as the number of iterations increased, the trend of normal force error would decrease, the total reward increased with the number of iterations, and the normal force error tended to be stable.

**Figure 11:**
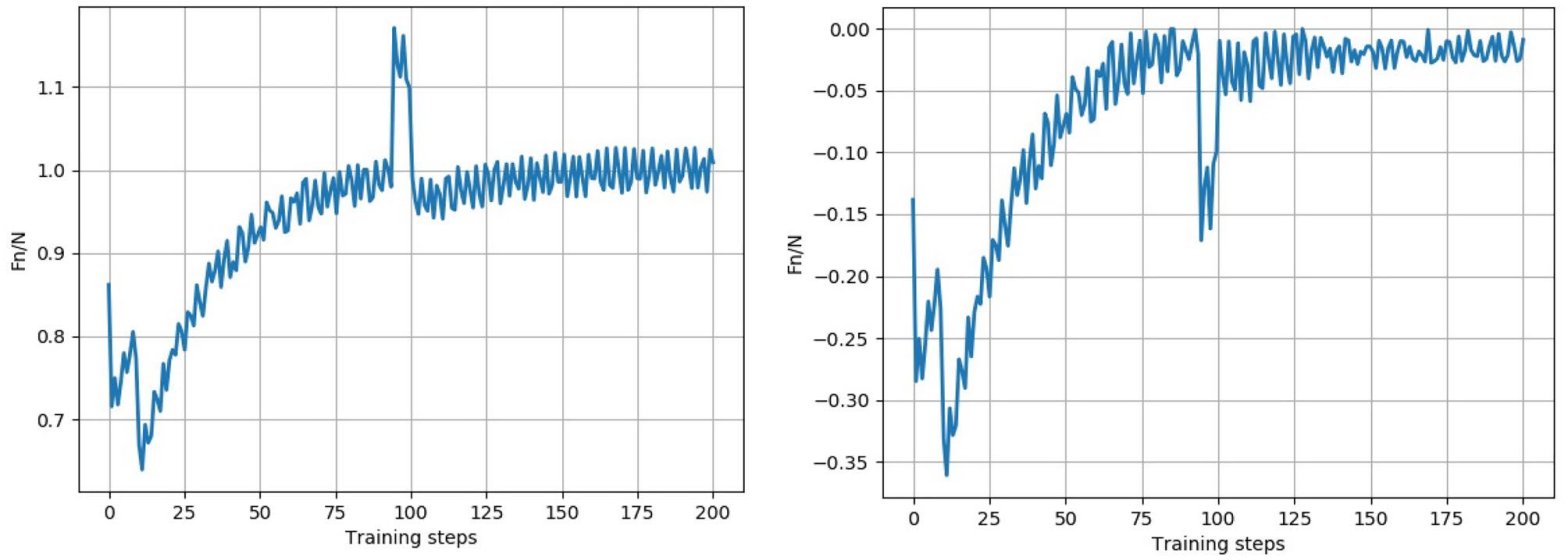
Plot of normal force variation and reward function variation for the SAC algorithm.

Taking a set of experimental results after convergence, the standard force change and reward function change for the converged experimental results are shown in Fig. 11. Comparing Fig. 11 with Fig. 8, it can be concluded that the controller compensation parameters found using the SAC algorithm can keep the normal force under control at the target normal force and can quickly adjust the normal force to the target normal force when there is a sudden change in force.

## Experimentation

**Figure 12:**
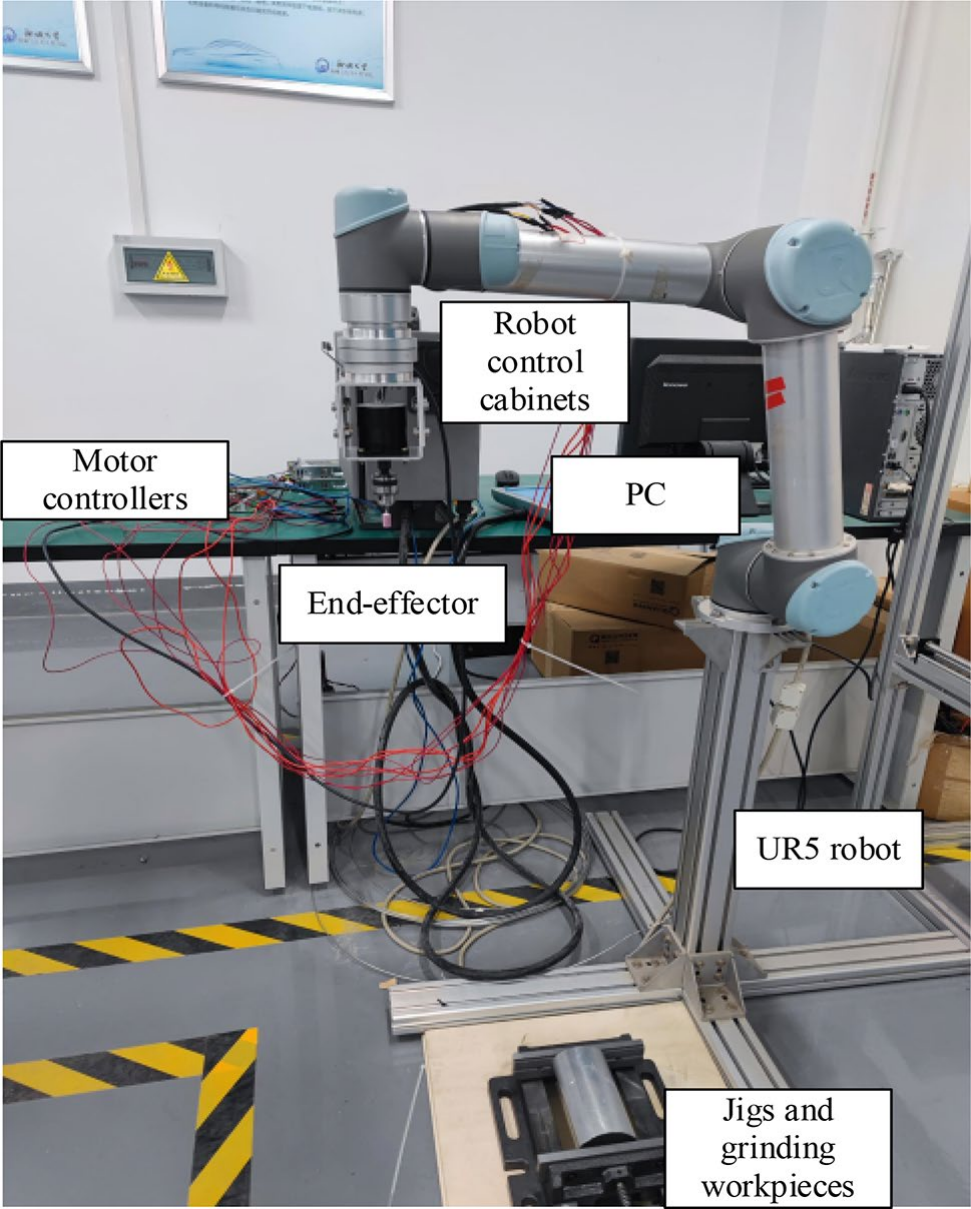
The robot constant force grinding experimental system platform.

**Figure 13:**
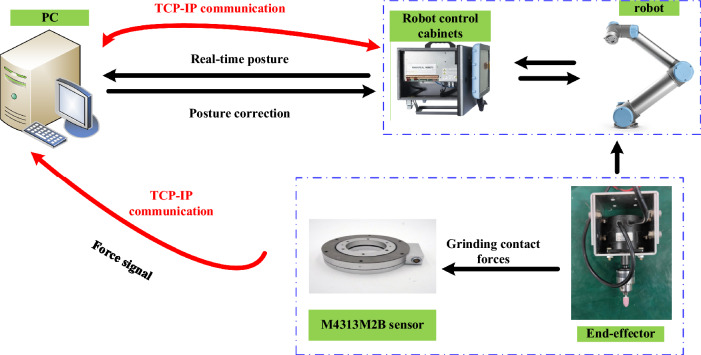
Diagram of a constant force solution for grinding control based on the UR5 robot.

To validate the robot constant force grinding intelligence algorithm, a robot constant force grinding experimental system platform is built. The platform consists of a UR5 robot, a robot control cabinet, force sensors, end-effectors, motor controllers, fixtures and grinding tools, and a PC, which performs the grinding task and the reading and processing of force information. The robot constant force grinding experimental system platform is shown in Fig. 12. The constant force grinding control scheme based on UR5 robot is shown in Fig. 13.

**Figure 14:**
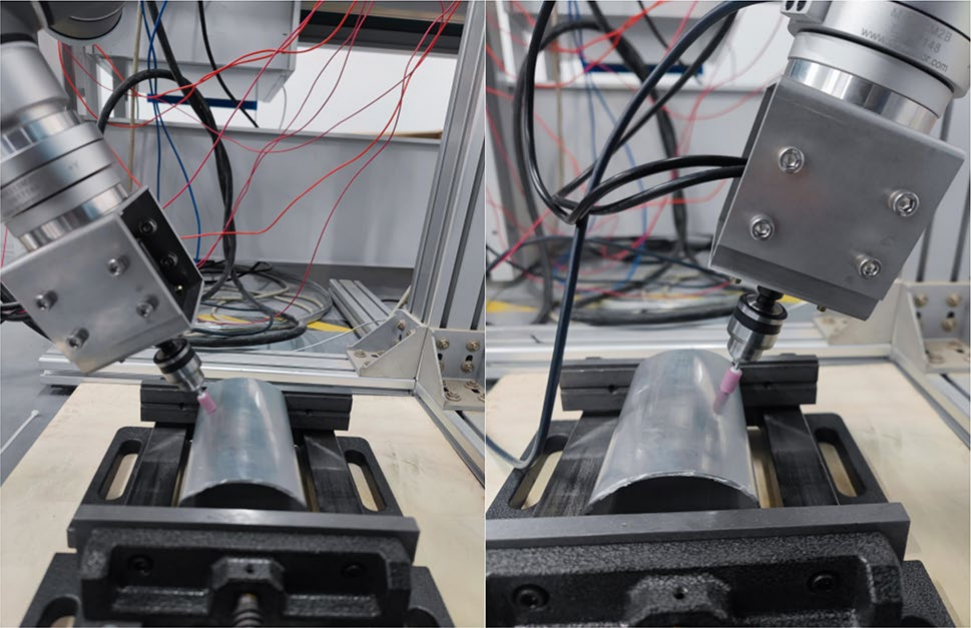
Robotic grinding process.

After fixing the workpiece with the fixture, the initial grinding trajectory was planned using the demonstrator, and the UR5 robot was controlled to move the end grinding device to the initial point of grinding. After the communication was established, the UR5 robot running algorithm was controlled using the host computer, and the speed of the grinding head was kept constant during the experiment. The sanding process is shown in Fig. 14.

During the experiments, the force sensor software was used to eliminate the effects of gravity and heavy moments, and the force data was subsequently synchronized into Python for coordinate conversion and Kalman filtering and processed into the algorithm.

In the experiment, the "adj zero" function in the six-dimensional force sensor acquisition software iDAS R&D is used to eliminate the influence of gravity and gravitational moment. Then the force data are synchronized into Python for coordinate conversion and Kalman filtering, and then into the algorithm after processing.

**Figure 15:**
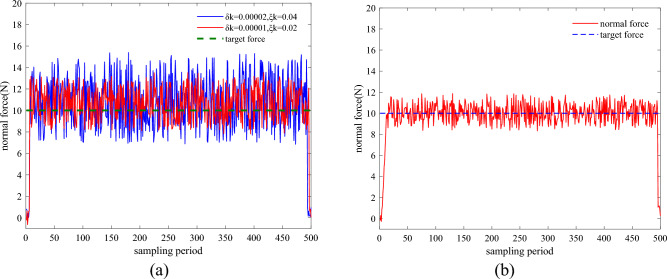
(**a**) Variation of grinding normal force using fixed compensation parameters. (**b**) Variation of normal force using the SAC algorithm.

To validate the SAC algorithm-based constant force grinding control, the Actor network in the trained SAC neural network model was used online to select the PID force controller compensation parameters when the robot end moved according to the initial trajectory, and the weights of the SAC algorithm neural network were trained offline using the experimental data when the grinding task was completed. To prevent the robot from being overloaded during the grinding process, the parameters of the force controller were restricted by setting the parameters in Eq. (2) $\delta_{k}$ = 0.00001, $\zeta_{k}$ = 0.02 and the range of compensation parameters to [0, 0.00002] and to [0, 0.04]. The compensation parameters were first set as fixed by taking to be 0.00001, 0.02 and 0.00002,0.04. Force controller robot intercepts the normal force variation plot during grinding at 0.04, and the variation plot is shown in Fig. 15a.

As can be seen from Fig. 15a, when the PID force controller uses fixed compensation parameters, the grinding normal force cannot be stabilized at the target grinding force of 10N during the grinding process and has large fluctuations, which cannot meet the requirements of constant force control. The robot was moved to the initial grinding point, fed according to the initial grinding trajectory, and the optimal controller compensation parameters were selected using the Actor network in the trained SAC algorithm. The optimal compensator parameters were applied to the force controller for the experiment. As shown in Fig. 15b, the normal grinding force gradually stabilized as the iterations of the SAC algorithm proceeded, and the final selection of the optimal controller compensation parameters allowed the force controller to remain on the target grinding force during grinding and maintain it within ± 2 N. Put the maximum, minimum, and average values of the data in Fig. 15a,b into Table 3. From Table 3, it can be seen that the maximum normal force using the SAC model is reduced by about 15% compared to the maximum normal force using fixed parameters, the minimum normal force is reduced by about 7%, and the average normal force is reduced by about 6%.

**Table 3:** the maximum, minimum, and average values of the data.

Normal force using using $\delta_{k}$ = 0.00001, $\zeta_{k}$ = 0.02	Normal forcer using SAC model	
Maximum value	13.4 N	11.3 N
Minimum value	8.2 N	8.8 N
Average value	10.7 N	10.02 N

The comparison between simulation and experimental data is shown in Table 4. Due to the small weight of the end effector during simulation, the target normal force during simulation was 1N. From Table 4, it can be seen that both the simulated and experimental normal forces achieved the target normal force, but the average value of the simulated normal force was smaller than the actual average value. This is because the experiment did not achieve the ideal simulation environment.

**Table 4:** Comparison of simulation and experimental data.

Simulation data	Experimental data	
Maximum value	1.15 N	11.3 N
Minimum value	0.65 N	8.8 N
Average value	1.00 N	10.02 N

**Figure 16:**
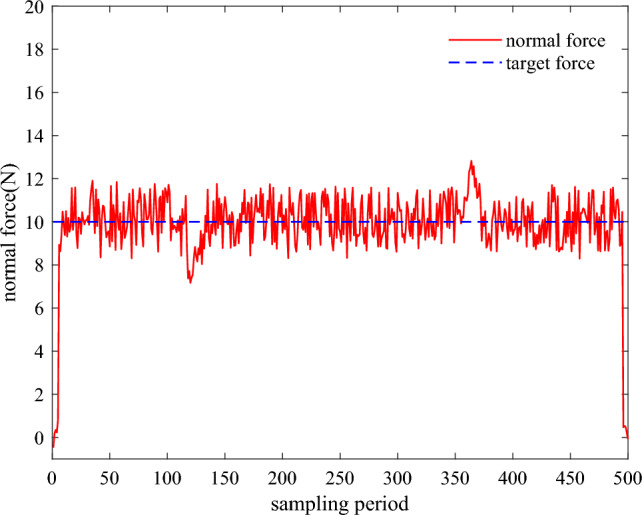
Plot of the change in normal force with the addition of protrusions and grooves.

To verify the algorithm's ability to adapt to the environment, protrusions and grooves on the ground workpiece are added to the grinding experiment, and the normal force change graph obtained is shown in Fig. 16. As can be seen in the figure, after hitting the interference during grinding, the normal force undergoes a sudden change, followed by a rapid adjustment to converge to the target normal force. Finally, it fluctuates on the target normal force, proving that the constant force grinding controller with the addition of the SAC algorithm, has a strong ability to adapt to the environment.

## Summary

This paper proposes an optimal parameter finding algorithm based on SAC to solve the problem that traditional PID controllers cannot control the robot to perform stable constant force grinding under environmental changes due to the difficulty of obtaining the compensation term parameters. Coppeliasim is used to build a robot grinding simulation model with force-aware information, and simulation experiments of chewing surfaces are carried out, which show that the constant force controller with this algorithm can maintain constant force when the robot grinds surfaces, and it is feasible. Finally, the experimental verification is carried out on the platform of the robot constant force-grinding experimental system. The results show that the optimal parameter finding algorithm based on SAC can help the PID controller to obtain the optimal compensation term. The PID controller with the optimal compensation term has a good effect on controlling the robot's constant force grinding with a strong ability for environmental adaptation. This article only deals with the comparison of PID controllers using fixed parameters and SAC algorithms. In the following, we will use other reinforcement learning grinding experiments to verify the advantages of reinforcement learning algorithms (Supplementary Information file 1).

## Supplementary Information

Supplementary Information.

## Data Availability

The datasets used during the current study available from the corresponding author on reasonable request.
